# *TMEM106B* haplotypes have distinct gene expression patterns in aged brain

**DOI:** 10.1186/s13024-018-0268-2

**Published:** 2018-07-03

**Authors:** Yingxue Ren, Marka van Blitterswijk, Mariet Allen, Minerva M. Carrasquillo, Joseph S. Reddy, Xue Wang, Thomas G. Beach, Dennis W. Dickson, Nilüfer Ertekin-Taner, Yan W. Asmann, Rosa Rademakers

**Affiliations:** 1 Department of Health Sciences Research, Mayo Clinic, Jacksonville, FL USA; 2 Department of Neuroscience, Mayo Clinic, Jacksonville, FL USA; 3 Civin Laboratory for Neuropathology, Banner Sun Health Research Institute, Sun City, AZ USA; 4 Department of Neurology, Mayo Clinic, Jacksonville, FL USA

**Keywords:** TMEM106B, Frontotemporal dementia, Co-expression networks, Differential expression, Synaptic transmission, Immune response, Progranulin

## Abstract

**Background:**

Single nucleotide polymorphisms (SNPs) inherited as one of two common haplotypes at the *transmembrane protein 106B* (*TMEM106B*) locus are associated with the risk of multiple neurodegenerative diseases, including frontotemporal lobar degeneration with pathological inclusions of TDP-43. Among the associated variants, rs3173615 (encoding p.T185S) is the only coding variant; however, non-coding variants may also contribute to disease risk. It has been reported that the risk haplotype is associated with higher levels of TMEM106B and increased levels of TMEM106B cause cytotoxicity; however, the precise mechanism through which *TMEM106B* haplotypes contribute to neurodegeneration is unclear.

**Methods:**

We utilized RNA sequencing data derived from temporal cortex (TCX) and cerebellum (CER) from 312 North American Caucasian subjects neuropathologically diagnosed with Alzheimer’s disease, progressive supranuclear palsy, pathological aging or normal controls to analyze transcriptome signatures associated with the risk (TT) and protective (SS) *TMEM106B* haplotypes. In cohorts matched for disease phenotype, we used Analysis of Variance (ANOVA) to identify differentially expressed genes and Weighted Gene Co-expression Network Analysis (WGCNA) to identify gene networks associated with the risk and protective *TMEM106B* haplotypes.

**Results:**

A total of 110 TCX and 116 CER samples were included in the analyses. When comparing TT to SS carriers, we detected 593 differentially expressed genes in TCX and 7 in CER. Gene co-expression network analyses further showed that in both TCX and CER the SS haplotype was positively correlated with gene networks involved in synaptic transmission, whereas the TT haplotype was positively correlated with gene networks enriched for immune response. Gene expression patterns of 5 cell-type-specific markers revealed significantly reduced expression of the neuronal marker and relative increases in all other cell markers in TT as compared to SS carriers in TCX with a similar but non-significant trend in CER.

**Conclusions:**

By comparing the common *TMEM106B* risk and protective haplotypes we identified significant and partly conserved transcriptional differences across TCX and CER and striking changes in cell-type composition, especially in TCX. These findings illustrate the profound effect of *TMEM106B* haplotypes on brain health and highlight the importance to better understand TMEM106B’s function and dysfunction in the context of neurodegenerative diseases.

**Electronic supplementary material:**

The online version of this article (10.1186/s13024-018-0268-2) contains supplementary material, which is available to authorized users.

## Background

Given the world’s aging population, neurodegenerative diseases have become a major cause of disability and death. While the disease mechanisms differ, neurodegeneration often results from the accumulation of misfolded aggregated proteins in different areas of the aging brain, and this process yields cell death and inflammatory damage in those brain regions [1]. Recent studies have revealed that two common haplotypes in transmembrane protein 106B (*TMEM106B*) are associated with risk of multiple neurodegenerative diseases, most notably with frontotemporal lobar degeneration with pathological inclusions of TDP-43 (FTLD-TDP) [2–4], progranulin (*GRN*)-related FTLD [3, 5], chromosome 9 open reading frame 72 (*C9ORF72*)-mediated FTLD [6, 7] and hippocampal sclerosis of aging [8, 9]. *TMEM106B* haplotypes were also shown to associate with the development of cognitive impairment in amyotrophic lateral sclerosis (ALS) [10] and with the presence of TDP-43 pathology in Alzheimer’s disease (AD) [11] and elderly individuals without FTLD [12]. The broad involvement of *TMEM106B* in neurodegenerative diseases makes it an important gene to characterize and a promising target for potential therapies.

As with most risk loci identified by genome-wide association studies (GWAS), the functional variant(s) in the *TMEM106B* locus associated with the reported associations remains elusive. Within the associated linkage disequilibrium (LD) block, rs3173615 is the only variant encoding an amino acid change from the more common, highly conserved, threonine (Thr185; risk allele) to a serine (Ser185; protective allele) at position 185. In vitro, the protective (Ser185) TMEM106B isoform was consistently expressed at lower levels than the risk (Thr185) TMEM106B isoform due to an increased rate of protein degradation, possibly resulting from changes in TMEM106B glycosylation [13]. In addition to the coding variant (rs3173615) numerous non-coding variants also differentiate the *TMEM106B* haplotypes and at least one of these variants (rs1990620) was suggested to affected higher-order chromatin architecture at the *TMEM106B* locus and changes in mRNA expression [14]. Indeed, expression studies in FTLD-TDP brains have shown increased *TMEM106B* mRNA levels in carriers of the risk haplotype [2]. Regardless of the identity of the specific functional variant(s), these findings suggest the presence of higher levels of TMEM106B in carriers of the risk haplotype and lower levels of TMEM106B in carriers of the protective haplotype. These findings are in line with cell biological studies which showed that increased TMEM106B levels were cytotoxic and led to an increase in lysosomal size and reduced lysosomal acidification [4]. Importantly, however, the specific mechanism by which *TMEM106B* haplotypes and changes in its expression contribute to neurodegeneration remains unknown.

In this study, we utilized RNA sequencing data of temporal cortex (TCX) and cerebellum (CER) samples from 312 North American Caucasian subjects to identify transcriptome signatures associated with the *TMEM106B* haplotypes. By comparing homozygote rs3173615 TT (risk) and SS (protective) carriers, we discovered differentially expressed genes in both TCX and CER regions, and identified shared gene co-expression networks between TCX and CER through which the *TMEM106B* haplotypes may contribute to brain function and brain health.

## Materials and methods

### Dataset description

We used RNA sequencing data of 268 TCX and 266 CER brain samples from 312 North American Caucasian subjects with neuropathological diagnosis of AD, progressive supranuclear palsy (PSP), pathologic aging (PA; defined as aging in nondemented elderly humans that is associated with moderate to marked cerebral amyloid deposition in the absence of significant neurofibrillary degeneration) [15] or elderly controls (CON) without clinically-significant neurodegenerative diseases [16]. The tissue processing, RNA extraction, RNA sequencing, quality control and data normalization were previously described [16, 17]. To differentiate individuals with the *TMEM106B* risk and protective haplotypes we used rs3173615 as the tagging variant. Genotypes of rs3173615 for the 312 individuals were extracted by PLINK using data generated from Illumina Omni2.5 BeadChips [17].

### Differential gene expression analysis

Conditional Quantile Normalization (CQN) was previously performed on the raw gene counts to correct for GC bias and gene length differences, and to obtain similar quantile-by-quantile distributions of gene expression levels across samples [17]. Based on the bi-modal distribution of the CQN normalized and log2-transformed reads per kb per million (RPKM) gene expression values, for both TCX and CER samples, protein-coding genes with average log2 RPKM > = − 1 in at least one haplotype group were considered expressed above detection threshold and were included in further analysis. Using this selection threshold, 16,868 genes were included for the TCX analysis and 14,994 were included for the CER analysis.

Differential gene expression analyses were performed using Partek Genomics Suite (Partek Inc., St. Louis, MO). Gene expression between the rs3173615 SS and TT individuals were compared using Analyses of Variance models (ANOVA), while correcting for RNA integrity number (RIN), age at death, sex and disease type. The selection of these covariates was based on the source of variation analyses from which factors with mean F ratio > 1.25 were considered confounding factors that needed to be adjusted for. The *Benjamini–Hochberg* procedure was performed to adjust for multiple testing and control false discovery rate (FDR). Differentially expressed genes (DEG) were defined by thresholds of |fold change| (FC) > = 1.5 and adjusted *p* value < 0.05. The significance of DEG overlap between TCX and CER was tested by calculating empirical *p*-value based on 100,000 simulations. Pathway analyses of differentially expressed genes were performed using MetaCore pathway analysis (Thomson Reuters) (Version 6.25).

### Weighted gene co-expression network analysis

To identify groups of genes that are correlated with the *TMEM106B* haplotype, we performed Weighted Gene Co-expression Network Analysis (WGCNA) [18] using residual expression values calculated from adjusting for RIN, age at death, sex, and disease type. Separate WGCNA analyses were performed for the TCX and CER datasets. Signed hybrid co-expression networks were built for both WGCNA analyses. For each set of genes, a pairwise correlation matrix was computed and an adjacency matrix was calculated by raising the correlation matrix to a power. Based on the relationships between power and scale independence, the power of 4 was chosen for the TCX dataset, and the power of 14 was chosen for CER dataset. We used hybrid dynamic tree cutting, a minimum module size of 40 genes, and a minimum height for merging modules at 0.25 for both TCX and CER. Each module was summarized by the first principal component of the scaled (standardized) module expression profiles (module eigengene). For each module, the module membership measure (MM) was defined as the correlation between gene-expression values and the module eigengene. Hub genes from relevant modules, which have the highest connectivity to other genes within the module, were selected using the WGCNA function “chooseTopHubInEachModule”. Each module was assigned a unique color identifier, and genes that did not fulfil these criteria for any of the modules were assigned to the gray module. To assess the correlation of modules to the *TMEM106B* protective (SS) haplotype, we defined the TT genotype as 0, and SS as 1. Modules significantly associated with TT or SS were annotated using WGCNA R function GOenrichmentAnalysis. Modules were also tested for enrichment of the respective DEG signatures using the anRichment R package.

## Results

### Selection of study population and basic characteristics

In our available dataset, we identified 77 individuals homozygous for the C allele at rs3173615 corresponding to Thr185 (16 AD, 29 PSP, 8 PA and 24 CON; further referred to as TT) and 65 individuals homozygous for the G allele at rs3171615 corresponding to Ser185 (26 AD, 16 PSP, 5 PA and 18 CON, further referred to as SS) in the TCX. In the CER dataset, 80 individuals were TT (21 AD, 29 PSP, 5 PA and 25 CON) and 64 individuals were SS (26 AD, 16 PSP, 6 PA and 16 CON). Since our cohort comprised samples from cases with different neurodegenerative disease as well as neuropathologically normal individuals, we further matched TT and SS study groups on pathological diagnosis and subsequently on sex and age at death, where possible. In sum, we included 55 TT and 55 SS TCX samples (*n* = 110) with equal numbers of AD, PSP, PA and CON in each group (Additional file 1: Table S1). In the CER, we selected 58 TT and 58 SS samples (*n* = 116) with equal numbers of AD, PSP, PA and CON in each group. The 110 TCX and 116 CER samples were from 133 individual subjects (93 individuals had both CER and TCX samples). The general characteristics of the TT and SS study groups included in the analyses are presented in Table 1. TT and SS individuals were of comparable composition in terms of sex and age at death (by study design) and postmortem interval (PMI), and were also found to be similar in terms of Braak stage and brain weight.

**Table 1:** Characteristics of the subjects included in this study

Traits	TT	SS	*P* value
Sex (male %)	0.41	0.55	NA
Age at death	81.79 ± 9.31	81.15 ± 9.10	0.69
Post-mortem interval	4.43 ± 5.95	5.29 ± 7.39	0.53
Braak stage	3.63 ± 1.89	3.78 ± 1.99	0.72
Brain weight (g)	1116.60 ± 133.42	1144.13 ± 158.82	0.37

Sex and Age at death are known for all 133 subjects; Braak stage and post-mortem interval (PMI) are known for 98 subjects and brain weight is known for 93 subjects

### Transcriptional differences between TMEM106B TT and SS carriers in TCX and CER

We first compared transcriptome-wide gene expression between TT and SS carriers. In the TCX dataset, we identified 593 DEGs between TT and SS carriers (Additional file 1: Table S2). In the CER dataset, we only identified 7 DEGs (Additional file 1: Table S3), none of which overlapped with the TCX DEGs. The large difference between TCX and CER suggests that the impact of *TMEM106B* is more prominent in a brain region affected by neurodegeneration. To further study potential similarities in the expression changes in CER and TCX we compared the top 500 genes with |FC| ≥ 1.2 between SS and TT ranked by unadjusted *p* value in the TCX and CER dataset, which showed significant overlap in genes (*n* = 28; *p* = 0.0008) (Additional file 1: Table S4). Most of these genes are involved in neurotransmitter formation and regulation and interestingly *GAD1* (FC_CER_ 1.44; FC_TCX_ 1.90), *GAD2* (FC_CER_ 1.49; FC_TCX_ 2.14) and *SLC32A1* (FC_CER_ 1.39; FC_TCX_ 2.25)*,* all critical for GABAergic neurotransmission, are among the shared genes.

Next we performed pathway enrichment analysis on the DEGs. In TCX, 9 out of the 10 top enriched gene ontology (GO) terms were related to synaptic signaling or cell communication (Table 2). In the CER, since only 7 genes passed the thresholds of |FC| > = 1.5 and adjusted *p* values < 0.05, we loosened the threshold to |FC| > = 1.2 and unadjusted p values < 0.05 to allow the study of sufficient genes in the pathway analysis. In CER, we found most top enriched GO processes to be related to immune response; however, signaling was also among the top enriched GOs (Table 3).

**Table 2:** Top 10 enriched GO terms from differentially expressed genes in TCX

GO Process	*P* value	FDR	# Genes
single organism signaling	1.801E-20	5.424E-17	253
signaling	1.986E-20	5.424E-17	253
cell communication	5.539E-20	1.008E-16	256
G-protein coupled receptor signaling pathway	3.527E-19	2.908E-16	74
chemical synaptic transmission	4.260E-19	2.908E-16	59
trans-synaptic signaling	4.260E-19	2.908E-16	59
synaptic signaling	4.260E-19	2.908E-16	59
anterograde trans-synaptic signaling	4.260E-19	2.908E-16	59
multicellular organismal process	3.899E-18	2.366E-15	297
cell-cell signaling	3.492E-17	1.907E-14	91

**Table 3:** Top 10 enriched GO terms from differentially expressed genes in CER

GO Process	P value	FDR	# Genes
immune system process	1.990E-19	1.005E-15	101
defense response	3.083E-17	7.783E-14	69
regulation of multicellular organismal process	1.448E-16	2.438E-13	122
immune response	4.750E-16	5.996E-13	61
inflammatory response	1.576E-15	1.591E-12	38
response to stimulus	6.918E-15	5.822E-12	224
response to lipid	4.419E-14	3.187E-11	61
single organism signaling	1.247E-13	7.520E-11	163
signaling	1.341E-13	7.520E-11	163
positive regulation of multicellular organismal process	5.047E-13	2.548E-10	76

### Co-expression network analyses reveals gene clusters significantly correlated with TMEM106B TT and SS carrier status in TCX and CER

**Fig. 1:**
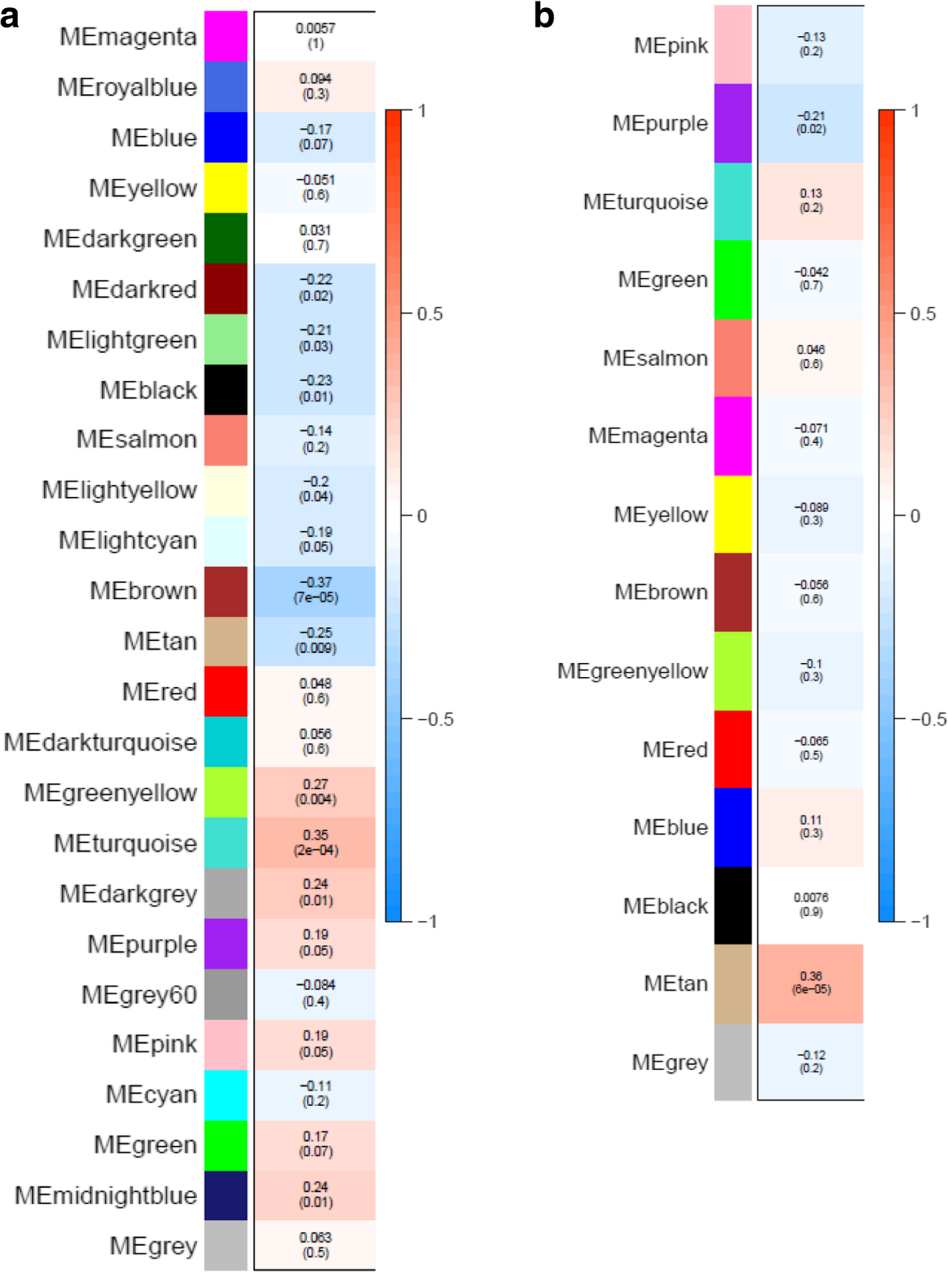
Module-haplotype correlation in (**a**) TCX and (**b**) CER. Each rectangle represents a module. The top number in each rectangle is the correlation coefficient (r) between the module eigengene to haplotype, and the bottom number is the p value of the correlation. The color orange represents positive correlation to the SS haplotype and blue represents negative correlation to SS (positive correlation to TT)

To gain further insight into the gene networks associated with the TMEM106B T (risk) and S (protection) haplotypes, we applied a gene co-expression network approach, WGCNA, which can functionally probe transcriptomic patterns of change. WGCNA is a computational tool that clusters genes in an unsupervised manner based on their correlated co-expression, and thus defines biologically relevant groups of genes that typically correspond to specific processes. WGCNA analysis of the TCX dataset identified 25 gene clusters (modules). The *TMEM106B* haplotype was significantly correlated with 10 modules: 4 positively correlated with SS and 6 positively correlated with TT (Table 4**,** Fig. 1a). WGCNA analysis of the CER dataset identified 14 modules. The *TMEM106B* haplotype was significantly correlated with 2 modules: 1 positively correlated with SS and 1 positively correlated with TT (Table 5**,** Fig. 1b). GO enrichment analysis on the modules significantly correlated with the *TMEM106B* haplotypes showed that synaptic transmission was a highly enriched GO in both TCX and CER from modules significantly positively correlated with the SS haplotype (modules turquoise in TCX and tan in CER). In addition, immune response was a highly enriched GO from modules significantly negatively correlated with the SS haplotype (positively correlated with TT) (modules darkred in TCX and purple in CER). This suggests that the *TMEM106B* haplotypes may influence brain function in multiple brain regions through similar mechanisms. Additionally, the TCX and CER synaptic transmission modules and the CER immune response module were significantly enriched for their respective DEGs, which demonstrated the consistency between the two analytic approaches (Additional file 1: Table S5).

**Table 4:** Modules significantly correlated to the *TMEM106B* rs3173615 status in TCX

Module	Correlation	P value	Top GO	FDR	Hub gene
turquoise	0.35	2e-04	synaptic transmission	6.64E-37	*FGF14*
greenyellow	0.27	0.004	Ribonucleoprotein complex biogenesis	1	*OXR1*
darkgrey	0.24	0.01	centriolar satellite	1	*IGSF9B*
midnightblue	0.24	0.01	ribosome binding	0.99	*TRAPPC2L*
brown	−0.37	7e-05	cell-cell adhesion	6.24E-07	*PDLIM5*
tan	−0.25	0.009	cellular response to zinc ion	0.025	*MFSD6*
black	−0.23	0.01	vasculature development	2.01E-19	*ADGRL4*
darkred	−0.22	0.02	humoral immune response	0.0002	*LIMK2*
lightgreen	−0.21	0.03	cytosolic ribosome	2.98E-123	*RPS19*
lightyellow	−0.2	0.04	extracellular matrix organization	9.04E-14	*DSP*

**Table 5:** Modules significantly correlated to the *TMEM106B* rs3173615 status in CER

Module	Correlation	P value	Top GO	FDR	Hub gene
tan	0.36	6e-05	synaptic transmission	4.74E-05	*PCP4L1*
purple	−0.21	0.02	immune response	1.42E-27	*ITGB2*

**Fig. 2:**
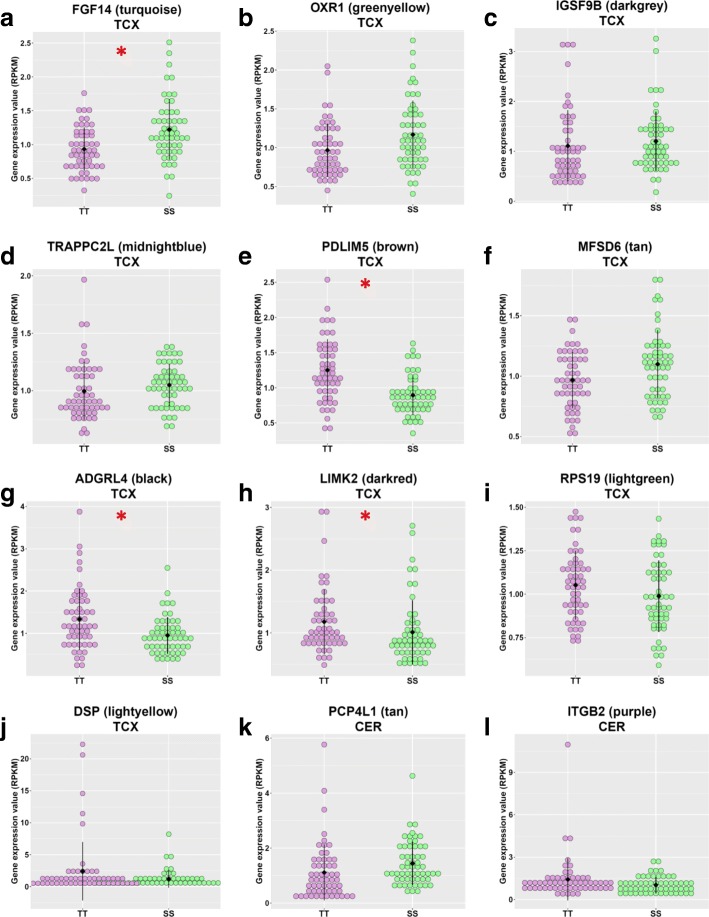
Expression values of the intramodular hub genes in significant modules in TT and SS carriers in TCX (**a**-**j**) and CER (**k**-**l**). The expression values (RPKM) have been adjusted for RIN, age at death, sex and disease type. The black dots represent the mean; the black lines represent the standard deviation. Red asterisks indicate significant difference between TT and SS (FDR <0.05)

**Fig. 3:**
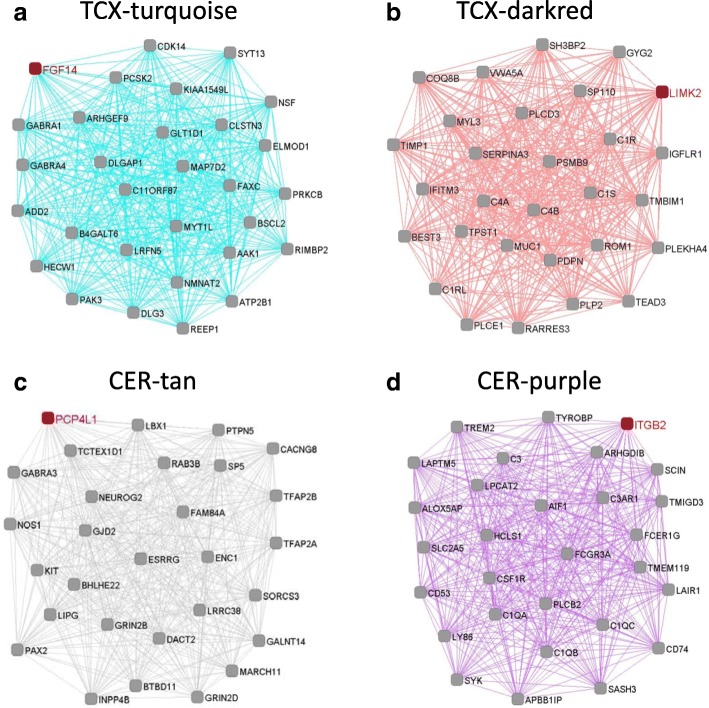
Interactions of top 30 highly connected genes in the synaptic transmission and immune response modules. **a** and **b** are modules in TCX. **c** and **d** are modules in CER. The red nodes represent hub genes of each module

We next identified the intramodular hub genes in the modules significantly correlated with the *TMEM106B* haplotype status. Hub genes are the genes with the highest connectivity to other genes of the same module; therefore, they often have important roles in the network and biological functions even if they do not meet the DEG threshold. Using the WGCNA function “chooseTopHubInEachModule”, we identified one hub gene for each significant module (Tables 5 and 6). The expression level of each hub gene in SS and TT carriers is shown in Fig. 2. For the synaptic transmission modules and immune response modules in TCX and CER, we also visualized the connection among hub genes and the top 30 genes with the highest connectivity within the respective modules using VisANT [19] (Fig. 3).

### Cell type composition may underlie expression differences observed in TMEM106B TT and SS carriers

**Fig. 4:**
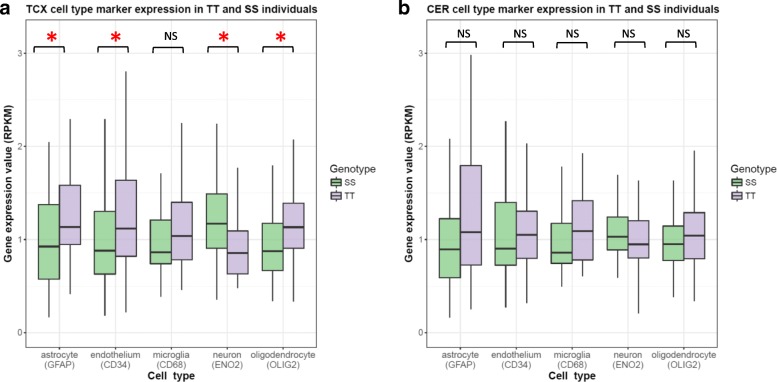
Expression values (RPKM) of the 5 cell-type marker genes. The expression values (RPKM) have been adjusted for RIN, age at death, sex and disease type. **a** TCX samples. **b** CER samples**.** Red asterisks represent significant difference between TT and SS (FDR < 0.05)

The central nervous system (CNS) includes 5 main cell types: neurons, astrocytes, microglia, oligodendrocytes, and endothelial cells. We hypothesized that the relative abundance of different cell types may at least partially explain the differences in the transcriptional signatures observed in TT and SS carriers. Because the cell type composition can be estimated by cell-type-specific gene expression levels, we adopted the approached described in Allen et al. [17] and used 5 genes to estimate the cell type composition in the brain tissue samples: *ENO2* for neurons (ENSG00000111674), *GFAP* for astrocytes (ENSG00000131095), *CD68* for microglia (ENSG00000129226), *OLIG2* for oligodendrocytes (ENSG00000205927), and *CD34* for endothelial cells (ENSG00000174059). When we compared the expression of these 5 genes in the TT and SS carriers we found, in TCX, that the SS carriers had significantly higher neuronal gene expression and lower astrocyte, endothelium and oligodendrocyte gene expression as compared to the TT carriers. The expression of the microglial marker CD68 was also lower in SS as compared to TT carriers, but this difference was not significant. In CER, the same trends were observed, although none of the differences were significant (Fig. 4).

## Discussion

In this study, we used RNA sequencing data of two brain regions to investigate the involvement of the *TMEM106B* risk and protective haplotypes in brain health. We identified large transcriptional differences between the protective SS and risk TT haplotypes in TCX, and much smaller differences in CER, consistent with the higher level of tissue damage and neuronal loss in the TCX region. Importantly, even though not statistically significant in CER, our data suggested similarities in the effects of *TMEM106B* haplotypes on the transcriptional signatures in both brain regions. By comparing the top 500 differentially expressed genes in TCX and CER with |fold change| ≥ 1.2 between SS and TT, we found 28 overlapping genes, all with a fold-change in the same direction (25 are increased and 3 are decreased in SS carriers as compared to TT carriers). Some of the biggest increases in SS carriers were seen in *GAD1* and *GAD2*, glutamic acid decarboxylases which are responsible for catalyzing the production of gamma-aminobutyric acid (GABA) from glutamate, and *SLC32A1* (also known as *VGAT*) which is a transporter involved in the uptake of GABA and glycine into synaptic vesicles. The specific changes in GABA-related signaling are interesting in light of a recent study which reported the preferential elimination of inhibitory synapses (defined by VGAT+ immunoreactivity) in the ventral thalamus of *Grn* knock-out mouse brains [20]. This observation raises the possibility that more robust GABAergic signaling or an increase in the number of inhibitory synapses at baseline in SS haplotype carriers may contribute to the profound protection conferred by this haplotype in patients with *GRN* mutations. Future immunohistochemical studies in human brain samples from SS and TT *TMEM106B* carriers are needed to confirm this hypothesis.

Using gene co-expression network analyses we further identified significant correlations of *TMEM106B* haplotypes with gene expression modules enriched for genes involved in similar biological processes across both brain regions. In TCX and CER, the *TMEM106B* SS haplotype was correlated with gene networks involved in synaptic transmission, whereas the TT haplotype was correlated with immune response networks in both brain regions, in addition to other specific gene networks such as cell-cell adhesion found only in TCX. In a further reference to the *Grn* knock-out mouse model, it is of interest to note that the gene expression modules most significantly correlated with the loss of *Grn* in cerebral cortex, cerebellum and hippocampus in mice were annotated by GO as involved in the innate immune response with *C1qa*, *C1qb*, *C1qc*, and *C3* among the most connected genes in the module, similar to what we observed when we compared SS to TT carriers in the CER (purple module; Fig. 3d) [20]. Finally, using 5 genes as surrogates for the 5 major brain cell types, we found that the gene networks were associated with the cell type composition in the TT and SS brains: the SS carriers showed higher neuronal gene expression as compared to the TT carriers, corresponding to an increase in networks related to synaptic transmission in SS brains; the TT carriers showed higher microglial gene expression as compared to SS carriers, in agreement with the observed enrichment for immune response networks in TT brains.

*TMEM106B* risk variants had previously been reported to be associated with both brain volume and connectivity. Specifically, using imaging studies, the *TMEM106B* risk allele was shown to significantly associate with reduced brain volume in non-demented elderly individuals, particularly in the superior temporal gyrus in the left hemisphere [21]. In addition, *GRN* mutation carriers with two copies of the *TMEM106B* risk allele demonstrated worsened brain connectivity compared to those who carried one or no risk alleles [22]. These authors found that *TMEM106B* haplotypes did not influence grey matter volume directly on its own, but in mutation carriers the protective *TMEM106B* haplotype was able to enhance the benefit of cognitive reserve on brain structure. Similarly, in our study, we found no significant differences in total brain weight between our TT and SS carriers. Instead our data suggest that the SS protective haplotype may confer higher synaptic transmission by strengthening brain connectivity in aged or diseased brain.

*TMEM106B* has also recently been linked to healthy neurological aging. In frontal cortex, the *TMEM106B* risk haplotype was associated with gene expression patterns suggestive of an older age than the individual’s true chronological age [23]. Similar to our study, the *TMEM106B* risk haplotype was further found to be associated with increased inflammation and reduced neuronal expression in these neuropathologically normal elderly individual. Contrary to our findings, however, they did not observe an effect of *TMEM106B* on gene expression in cerebellar tissues. This may be related to the fact that they focused on neurologically normal individuals whereas we included a mixture of neurodegenerative diseases and normal controls. To determine whether the significant modules we identified were mainly contributed by disease samples or controls, we performed additional WGCNA analyses with only cases in TCX (*n* = 74, 37 TT and 37 SS) or CER (*n* = 84, 42 TT and 42 SS), and using only controls in TCX (*n* = 42: 24 TT and 18 SS) or CER (*n* = 41: 25 TT and 16 SS). In both TCX and CER, analysis of the cases identified similar modules as we did in the full TCX and CER datasets (Additional file 1: Table S6). Furthermore, independent analyses of each disease group: TCX-AD, TCX-PSP, CER-AD and CER-PSP all identified synaptic transmission modules that are significantly correlated with the *TMEM106B* haplotype (Additional file 1: Table S7), suggesting that the effects of the *TMEM106B* haplotype was not specific to disease type. Analysis of only the control samples in TCX and CER identified less modules, and synaptic transmission and immune response modules were not significantly correlated with the *TMEM106B* haplotype in either brain region (Additional file 1: Table S8). While the smaller sample size of the control cohorts may have reduced our power to detect significant difference, these results suggest that the effects of *TMEM106B* haplotypes on the transcriptome are more pronounced in disease tissues than in healthy tissues.

## Conclusions

In summary, our study demonstrates significant and partly conserved effects of the *TMEM106B* haplotypes on the transcriptome across multiple brain regions. It is quite remarkable that a variant which is common in the normal population (minor allele frequency = 0.40) can have such profound effects on gene expression and cell type composition, especially since our SS and TT study groups were matched for disease phenotypes and age at death. This suggests that additional genetic and/or environmental factors are likely to modulate the effect of the *TMEM106B* haplotypes on neurological and normal brain aging in individual human subjects. TMEM106B has only begun to be characterized since the discovery of its association to FTLD-TDP several years ago. While its function has mostly been linked to lysosome functions and trafficking, and recently to myelination [24], our results suggest that *TMEM106B* plays a broader role in the CNS response to pathological or age-related insults in multiple brain regions. Continued research into *TMEM106Bs* normal function and dysfunction in the context of neurodegenerative diseases will be critical and holds promise for future therapeutic strategies.

## Additional file

Additional file 1: **Table S1.** Tissue samples available and selected for inclusion in this study. **Table S2.** DEGS in TCX. Positive fold change represents higher gene expression in SS than TT. Negative fold change represents lower gene expression in SS than TT. **Table S3.** DEGS in CER. Positive fold change represents higher gene expression in SS than TT. Negative fold change represents lower gene expression in SS than TT. **Table S4.** Overlapping genes between TCX and CER based on top 500 genes with |FC| ≥ 1.2 ranked by unadjusted *p* value. **Table S5.** Enrichment of modules for their respective DEG signatures. **Table S6**. Significant modules identified in the TCX and CER matched cases. **Table S7**. Significant modules identified in separate disease groups in TCX and CER. **Table S8**. Significant modules identified in the TCX and CER controls. (DOCX 54 kb)

## Abbreviations

AD: Alzheimer’s disease
ALS: Amyotrophic lateral sclerosis
AMP-AD: Accelerating Medicines Partnership in Alzheimer’s Disease
ANOVA: Analyses of variance
C9ORF72: Chromosome 9 open reading frame 72
CER: Cerebellum
CNS: Central nervous system
CON: Elderly controls
CQN: Conditional Quantile Normalization
DEG: Differentially expressed genes
FC: Fold change
FDR: False discovery rate
FTLD: Frontotemporal lobar degeneration
FTLD-TDP: Frontotemporal lobar degeneration with pathological inclusions of TDP-43
GO: Gene ontology
GRN: Progranulin
GWAS: Genome-wide association studies
LD: Linkage disequilibrium
MM: Module membership
PA: Pathologic aging
PMI: Postmortem interval
PSP: Progressive supranuclear palsy
RIN: RNA integrity number
RPKM: reads per kb per million
TCX: Temporal cortex
TMEM106B: Transmembrane protein 106
WGCNA: Weighted Gene Co-expression Network Analysis
