# Supplementary material for: TMEM106B haplotypes have distinct gene expression patterns in aged brain
Source: Mol Neurodegener. 2018 Jul 3;13:35. doi: 10.1186/s13024-018-0268-2 (PMC6029036; doi:10.1186/s13024-018-0268-2)
Supplement: Supplementary file 1 — Table S1. Tissue samples available and selected for inclusion in this study. Table S2. DEGS in TCX. Positive fold change represents higher gene expression in SS than TT. Negative fold change represents lower gene expression in SS than TT. Table S3. DEGS in CER. Positive fold change represents higher gene expression in SS than TT. Negative fold change represents lower gene expression in SS than TT. Table S4. Overlapping genes between TCX and CER based on top 500 genes with |FC| ≥ 1.2 ranked by unadjusted p value. Table S5. Enrichment of modules for their respective DEG signatures. Table S6. Significant modules identified in the TCX and CER matched cases. Table S7. Significant modules identified in separate disease groups in TCX and CER. Table S8. Significant modules identified in the TCX and CER controls. (DOCX 54 kb) [file 13024_2018_268_MOESM1_ESM.docx]

**Table S1. Tissue samples available and selected for inclusion in this study.**

|  | **TCX** | | | |  | **CER** | | | |
| --- | --- | --- | --- | --- | --- | --- | --- | --- | --- |
|  | **TT** | **SS** | **TT selected** | **SS selected** |  | **TT** | **SS** | **TT selected** | **SS selected** |
| **AD** | 16 | 26 | 16 | 16 |  | 21 | 26 | 21 | 21 |
| **PSP** | 29 | 16 | 16 | 16 |  | 29 | 16 | 16 | 16 |
| **PA** | 8 | 5 | 5 | 5 |  | 5 | 6 | 5 | 5 |
| **CON** | 24 | 18 | 18 | 18 |  | 25 | 16 | 16 | 16 |
| **Total** | 77 | 65 | 55 | 55 |  | 80 | 64 | 58 | 58 |

**Table S2. DEGS in TCX.** Positive fold change represents higher gene expression in SS than TT. Negative fold change represents lower gene expression in SS than TT.

| **Gene name** | **FDR** | **Fold change** |
| --- | --- | --- |
| *HECW1* | 0.00220957 | 1.53696 |
| *SLC7A2* | 0.0063307 | -1.64599 |
| *CAMKK1* | 0.000787552 | 1.58164 |
| *PDK4* | 0.00361976 | -1.67379 |
| *TAC1* | 0.0215311 | 1.63543 |
| *DLX6* | 0.00034143 | 1.9197 |
| *MAP3K9* | 0.00211965 | 1.51142 |
| *TTC22* | 0.00180202 | 1.50596 |
| *NOS2* | 0.000484172 | 1.60208 |
| *PAX7* | 0.00134998 | 1.76084 |
| *CD9* | 0.00593149 | -1.54978 |
| *CD22* | 0.028869 | -1.53896 |
| *CLCA4* | 0.0183909 | -1.5719 |
| *IGF1* | 0.00337053 | 1.64541 |
| *ATP1A2* | 0.00436628 | -1.59661 |
| *CYP24A1* | 0.0274701 | 1.59467 |
| *SYT13* | 0.00846062 | 1.52502 |
| *GABRA1* | 0.00323897 | 1.68791 |
| *VIM* | 0.00373246 | -1.52259 |
| *CD44* | 0.0274701 | -1.70187 |
| *ANK1* | 3.10E-05 | 1.92758 |
| *STAP1* | 0.00050535 | 2.10661 |
| *VCAN* | 0.00142645 | -1.63382 |
| *CDH1* | 0.00545497 | -1.5923 |
| *TNC* | 0.00406025 | -2.22325 |
| *CP* | 0.0120834 | -1.58195 |
| *EPN3* | 0.000342236 | 1.7824 |
| *FSTL4* | 0.00282016 | 1.59985 |
| *FOXC1* | 0.01066 | -1.51063 |
| *CBLN4* | 0.00167506 | 1.98536 |
| *STYK1* | 0.00202049 | 1.77679 |
| *LTK* | 0.000772599 | 1.69621 |
| *DMRT3* | 0.00130118 | 2.45193 |
| *CHI3L2* | 0.0234639 | -1.9746 |
| *GLP2R* | 0.0033048 | 1.71299 |
| *ERBB3* | 0.0125357 | -1.52774 |
| *TIE1* | 0.00223553 | -1.53145 |
| *CD84* | 0.0274797 | -1.50368 |
| *SYT1* | 0.0137388 | 1.51598 |
| *ATP2B3* | 0.00324414 | 1.51797 |
| *PTPN3* | 0.00481121 | 1.60272 |
| *CNGB1* | 0.0291169 | 1.61804 |
| *CYBRD1* | 0.00114504 | -1.5985 |
| *CDH19* | 0.021877 | -1.55094 |
| *FCGR2B* | 0.0169441 | -1.73295 |
| *MRVI1* | 0.00162266 | -1.55865 |
| *IPCEF1* | 0.00475321 | 1.52822 |
| *WSCD2* | 0.000406932 | 1.6479 |
| *PAK3* | 0.00351073 | 1.55768 |
| *AMPH* | 0.00228193 | 1.57024 |
| *SLC1A3* | 0.00596125 | -1.64328 |
| *SCGN* | 0.00127658 | 1.62972 |
| *CDH7* | 0.0306008 | 1.52163 |
| *LRP2* | 0.0288423 | -1.67797 |
| *PGR* | 0.011179 | -1.50689 |
| *ABCB1* | 0.00440869 | -1.57168 |
| *PTHLH* | 0.000656445 | 1.65381 |
| *RGS1* | 0.0445686 | -1.91276 |
| *SLC17A6* | 0.00127186 | 1.90308 |
| *CCDC80* | 0.01464 | -1.53401 |
| *MYH7* | 0.0017561 | 1.54043 |
| *TDRD1* | 0.000400729 | 2.08352 |
| *NEFH* | 0.0241827 | 1.50282 |
| *RASD2* | 0.000369536 | 1.52064 |
| *PVALB* | 0.00702668 | 1.87207 |
| *BDKRB1* | 0.00796982 | 1.73397 |
| *HNF4A* | 0.00199733 | 1.89963 |
| *NKAIN4* | 0.0024006 | -1.54247 |
| *EEF1A2* | 0.00537634 | 1.51281 |
| *SLC32A1* | 0.000331357 | 2.24779 |
| *SRPX* | 0.00539701 | -1.54441 |
| *SYTL4* | 0.00539668 | -1.68193 |
| *HTR2A* | 0.00666435 | 1.51587 |
| *ACP5* | 0.00521953 | -1.51326 |
| *FGF9* | 0.00104435 | 1.66318 |
| *SGCG* | 0.000614747 | 1.72091 |
| *FLT1* | 0.0231772 | -1.52446 |
| *OLFM4* | 0.00202082 | 1.73598 |
| *CBLN1* | 3.10E-05 | 1.83166 |
| *CRYM* | 0.0195463 | 1.53763 |
| *RASL12* | 0.00551573 | -1.68054 |
| *RHOV* | 0.00152947 | 1.62892 |
| *CA2* | 0.00155656 | -1.55878 |
| *TRPA1* | 0.00180202 | 1.57243 |
| *CALB1* | 0.0026889 | 1.66518 |
| *ESRP1* | 0.00682049 | 1.5734 |
| *NCALD* | 0.00572307 | 1.52555 |
| *TNFRSF10A* | 0.00453351 | -1.533 |
| *HAS1* | 0.00109887 | 1.53499 |
| *THEG* | 0.0431759 | 1.51137 |
| *ATP4A* | 0.0189052 | 1.61391 |
| *DLX5* | 0.000764976 | 1.8237 |
| *MET* | 0.000423654 | 1.87916 |
| *WNT2* | 0.00115878 | 1.62814 |
| *CPED1* | 0.029476 | -1.50864 |
| *CRHR2* | 0.000874755 | 1.58557 |
| *GALNTL5* | 0.00062405 | 1.58832 |
| *ECM2* | 0.0105719 | -1.67864 |
| *KANK1* | 0.000191301 | -1.53423 |
| *ATRNL1* | 0.00453337 | 1.5137 |
| *PKD2L1* | 0.00144923 | 1.75615 |
| *ASPA* | 0.0195172 | -1.55615 |
| *CACNG1* | 0.0162874 | 1.65956 |
| *GABRA4* | 0.00455065 | 1.55109 |
| *CRYAB* | 0.0289099 | -1.53741 |
| *KIAA1549L* | 0.00491893 | 1.52689 |
| *SLC1A2* | 0.0200938 | -1.57876 |
| *CD5* | 0.000570628 | 1.53923 |
| *ELMOD1* | 0.00153885 | 1.58606 |
| *VWF* | 0.00355837 | -1.64927 |
| *CUX2* | 0.00169167 | 1.73591 |
| *KCNA1* | 0.00268084 | 1.53346 |
| *FHL5* | 0.0316729 | -1.53221 |
| *VNN2* | 0.0136121 | -1.88791 |
| *SLC16A10* | 0.000304471 | 1.53638 |
| *PRSS16* | 0.00373515 | 1.54187 |
| *C7* | 0.0433954 | -1.61987 |
| *CDH9* | 0.00596119 | 1.55284 |
| *SPARC* | 0.00340196 | -1.51443 |
| *RASGRF2* | 0.000130196 | 1.52686 |
| *GABRG2* | 0.00346383 | 1.71774 |
| *NPR3* | 0.000342236 | 2.06696 |
| *HRH2* | 0.000821001 | 1.64623 |
| *FGF12* | 0.00313114 | 1.51142 |
| *RTKN* | 0.00877206 | -1.50319 |
| *TLX2* | 0.000570628 | 1.70015 |
| *TACR1* | 0.000512531 | 1.74334 |
| *EFEMP1* | 0.00652818 | -1.62123 |
| *IL1R2* | 0.0221291 | -2.23869 |
| *IL1R1* | 0.0102076 | -1.51172 |
| *IL18R1* | 0.0373641 | -1.63968 |
| *DLX2* | 0.000191301 | 1.71865 |
| *EPHA4* | 0.00320754 | 1.50558 |
| *OPRD1* | 0.00407303 | 1.62667 |
| *HAO2* | 0.00568688 | 1.57116 |
| *ID3* | 0.00378131 | -1.53959 |
| *HSD11B1* | 0.0088916 | -1.53963 |
| *OLFM3* | 0.016994 | 1.5635 |
| *ABCG2* | 0.00623812 | -1.5721 |
| *SPP1* | 0.00874728 | -1.84259 |
| *PPL* | 0.000471101 | 1.64084 |
| *TJP2* | 0.000901392 | -1.60696 |
| *ONECUT2* | 0.00222012 | 1.86734 |
| *PRLHR* | 0.00907888 | 1.72114 |
| *DUSP1* | 0.00104286 | -1.58732 |
| *CHRNA2* | 0.000342236 | 2.10659 |
| *SV2C* | 0.00223581 | 2.15737 |
| *FLT3* | 0.00739214 | 1.70801 |
| *NBPF14* | 0.00817918 | -1.51666 |
| *SRGN* | 0.00672357 | -1.55918 |
| *SSPN* | 0.00115547 | -1.52172 |
| *PDE1B* | 0.00138921 | 1.5041 |
| *NFE2* | 0.0456859 | -1.51364 |
| *PLP1* | 0.0249384 | -1.54185 |
| *GPR83* | 0.00593774 | 1.75376 |
| *KCNS1* | 0.00247028 | 1.7054 |
| *KCNK15* | 0.00202936 | 1.658 |
| *MYRF* | 0.016104 | -1.55546 |
| *AHNAK* | 0.00414155 | -1.62204 |
| *NPBWR2* | 0.000499848 | 2.11225 |
| *NAPB* | 0.00275649 | 1.51839 |
| *GFRA4* | 0.0303107 | 1.55064 |
| *LAMP5* | 0.00138159 | 1.94279 |
| *LRRN4* | 0.0019568 | 1.50071 |
| *GRPR* | 0.0490022 | 1.52445 |
| *EVI2A* | 0.0200602 | -1.58526 |
| *OMD* | 0.0129397 | -1.76294 |
| *ATP13A4* | 0.00509634 | -1.51963 |
| *KLF2* | 0.0185388 | -1.50246 |
| *GNG13* | 0.00285879 | 1.50406 |
| *YWHAH* | 0.00600416 | 1.52418 |
| *RFPL1* | 0.0101403 | 1.51875 |
| *MCHR1* | 0.00104384 | 1.74646 |
| *GAD1* | 0.000742515 | 1.90105 |
| *KCNC1* | 0.00135269 | 1.59343 |
| *GFAP* | 0.00568688 | -1.54391 |
| *GALNT15* | 0.00241223 | -1.6873 |
| *ITGB4* | 0.00253661 | -1.84088 |
| *SSTR4* | 0.00110743 | 1.98254 |
| *ATP1A4* | 0.0227797 | -1.55386 |
| *SYT4* | 0.0163339 | 1.51257 |
| *ATP8A2* | 0.00166066 | 1.59217 |
| *MTUS2* | 0.000421181 | 1.68905 |
| *ALOX5AP* | 0.0272882 | -1.51405 |
| *CHRM3* | 0.0043681 | 1.54236 |
| *MRO* | 0.00275074 | -1.6602 |
| *NOTCH2* | 0.00896436 | -1.50053 |
| *FST* | 0.00129152 | 1.66285 |
| *EMP1* | 0.00739168 | -1.63358 |
| *C12orf39* | 0.0011691 | -1.54763 |
| *LRP4* | 0.0011202 | -1.51018 |
| *CYP2J2* | 0.00032645 | -1.51676 |
| *APLNR* | 0.00552162 | -2.03854 |
| *TESPA1* | 0.00918893 | 1.62282 |
| *AGT* | 0.0054389 | -1.53223 |
| *RGS8* | 0.00463875 | 1.73917 |
| *PCDH8* | 0.0023272 | 1.92211 |
| *EDNRB* | 0.0057031 | -1.52266 |
| *DGKB* | 0.00375668 | 1.56682 |
| *GAD2* | 0.00077876 | 2.13815 |
| *KLF4* | 0.00487948 | -1.63253 |
| *TLR4* | 0.00128177 | -1.70536 |
| *ENPP2* | 0.0129998 | -1.54618 |
| *IFNA21* | 0.00843459 | 1.69139 |
| *GMPR* | 0.0112123 | -1.52897 |
| *LRRC32* | 0.0105859 | -1.52058 |
| *YAP1* | 0.00509634 | -1.5202 |
| *STAT4* | 0.00255285 | 1.71402 |
| *HCN4* | 0.00152947 | 1.53037 |
| *AGPAT9* | 0.000193641 | 1.61006 |
| *PDE6H* | 0.0247768 | 1.50141 |
| *SLCO1C1* | 0.0135898 | -1.53172 |
| *KCNH5* | 0.00840185 | 1.607 |
| *ADAD2* | 0.00278162 | 1.60293 |
| *ABCA8* | 0.00391205 | -1.76283 |
| *SLC14A1* | 0.00590599 | -1.92605 |
| *TTYH2* | 0.00387245 | -1.60229 |
| *CBLN2* | 0.0021738 | 1.54237 |
| *PMAIP1* | 0.0101454 | -1.54906 |
| *GRB7* | 0.00034143 | 1.86777 |
| *HUNK* | 0.00077876 | 1.69166 |
| *CYR61* | 0.0142839 | -1.50024 |
| *CYP4B1* | 0.017308 | -1.89535 |
| *IGSF3* | 0.000146653 | 1.65359 |
| *XCL1* | 0.0475019 | 1.51237 |
| *FAM163A* | 0.000369536 | 2.1932 |
| *KCNH1* | 0.00112329 | 1.82883 |
| *KCNN3* | 0.000471101 | -1.66444 |
| *PKLR* | 0.0114112 | 1.5645 |
| *SYT2* | 0.00590599 | 1.67654 |
| *FBLN7* | 0.000627712 | 1.81792 |
| *SCN1A* | 0.00128195 | 1.53423 |
| *SLC4A10* | 0.00441375 | 1.60087 |
| *DLX1* | 0.000643423 | 1.73357 |
| *PTH2R* | 0.00161003 | 1.67319 |
| *NYAP2* | 0.00216959 | 1.6385 |
| *ACKR3* | 0.00269075 | -1.52923 |
| *42798* | 0.000959325 | 1.63965 |
| *ALDH1L1* | 0.00674001 | -1.50867 |
| *SLC10A4* | 0.000511605 | 1.75235 |
| *CDH18* | 0.00135696 | 1.78129 |
| *MYO10* | 0.000570628 | -1.50744 |
| *HAPLN1* | 0.00442232 | 1.59146 |
| *LIX1* | 0.00529286 | -1.57405 |
| *MEGF10* | 0.000363821 | -1.55571 |
| *C1QTNF2* | 0.000120534 | 1.57388 |
| *GABRA6* | 0.00484185 | 1.72353 |
| *GABRB2* | 0.00516122 | 1.59726 |
| *CPLX2* | 0.0096273 | 1.54669 |
| *TPBG* | 0.000164538 | 1.72837 |
| *PRSS35* | 0.0135575 | -1.6263 |
| *FAXC* | 0.00165913 | 1.5125 |
| *VIP* | 0.00520015 | 1.70901 |
| *FERD3L* | 0.0423349 | 1.52171 |
| *LANCL3* | 0.0011395 | 1.53419 |
| *LPAR4* | 0.0256048 | -1.59638 |
| *FATE1* | 0.0282209 | 1.56529 |
| *CHRNA6* | 0.000342236 | 1.93989 |
| *DOCK5* | 0.00538953 | -1.57247 |
| *STAR* | 0.000584953 | 1.55909 |
| *ST18* | 0.0212761 | -1.53293 |
| *CRH* | 0.00674001 | 2.21183 |
| *RSPO2* | 0.00123656 | 1.65723 |
| *MAL2* | 0.0130375 | 1.50674 |
| *NTRK2* | 0.00296947 | -1.51701 |
| *SLC39A12* | 0.0101676 | -1.54935 |
| *HABP2* | 0.00263812 | 1.50458 |
| *HTR3B* | 0.000455736 | 1.94405 |
| *CDH22* | 0.00066587 | 1.73578 |
| *CNTN5* | 0.00131797 | 1.73601 |
| *LYPD6B* | 0.000517825 | 1.75644 |
| *CNDP1* | 0.0063197 | -1.54595 |
| *SLC7A11* | 0.0077487 | -1.51591 |
| *GPR158* | 0.00286731 | 1.60182 |
| *BTBD11* | 0.000130196 | 1.85957 |
| *PTPRO* | 0.000300094 | 1.68781 |
| *ZNF385D* | 0.000977977 | 1.51499 |
| *SLC35F4* | 0.00310501 | 1.6188 |
| *GLT1D1* | 0.00146986 | 1.57675 |
| *TMEM132D* | 0.00152383 | 1.50281 |
| *MCHR2* | 0.0259922 | 1.56879 |
| *MEPE* | 0.00114261 | 2.1461 |
| *GJA1* | 0.00582146 | -1.81456 |
| *CCNO* | 0.000511605 | 1.87858 |
| *BMP3* | 0.0119114 | 1.50847 |
| *IFLTD1* | 0.00578769 | -1.76013 |
| *BMP6* | 0.00514763 | -1.60661 |
| *PTPRR* | 0.00398732 | 1.54885 |
| *CDH12* | 0.00216959 | 1.59154 |
| *ANGPT1* | 0.00246864 | -1.52407 |
| *GPR26* | 0.00157756 | 1.68643 |
| *C10orf90* | 0.0103239 | -1.50398 |
| *C21orf91* | 0.0157202 | -1.5582 |
| *APCDD1* | 0.00174329 | -1.51075 |
| *PIEZO2* | 0.0352853 | -1.5439 |
| *SLC16A1* | 0.00175113 | -1.67363 |
| *ADAMTS3* | 0.0017561 | 1.56524 |
| *SH3RF2* | 0.00110743 | 1.59268 |
| *KCNS2* | 0.00158738 | 1.77476 |
| *UNC5D* | 0.0049334 | 1.52565 |
| *LHFPL4* | 0.000614747 | 1.63204 |
| *BUB1B* | 0.00142645 | 1.59488 |
| *NMNAT2* | 0.00300818 | 1.56016 |
| *HTR5A* | 0.00671727 | 1.57577 |
| *KIT* | 0.0013367 | 1.54529 |
| *KCNJ6* | 0.0016807 | 1.55453 |
| *KCNB1* | 0.00144923 | 1.5238 |
| *ADAMTS4* | 0.0457849 | -1.51085 |
| *WNT9B* | 0.00095456 | 1.51151 |
| *LAD1* | 0.00928996 | 1.54842 |
| *GJD2* | 0.000342236 | 1.9986 |
| *CALM3* | 0.00284346 | 1.53635 |
| *S100B* | 0.00761112 | -1.51518 |
| *HIPK4* | 0.00116501 | 1.5772 |
| *CHRNB2* | 0.00241223 | 1.57642 |
| *AZGP1* | 0.014451 | -1.66904 |
| *IP6K3* | 0.0371474 | -1.60312 |
| *GNG3* | 0.00246504 | 1.59323 |
| *LRRC38* | 0.00297126 | 1.64127 |
| *FCGR3B* | 0.0161324 | -1.81579 |
| *FAM84A* | 0.00297176 | 1.53138 |
| *KCNJ3* | 0.000538322 | 1.60234 |
| *NEUROD1* | 0.0177412 | 1.6004 |
| *VSNL1* | 0.0131378 | 1.5636 |
| *TEKT4* | 0.00614467 | 1.91844 |
| *S100A9* | 0.0151801 | -2.19669 |
| *CXCR1* | 0.0130375 | -2.27157 |
| *TGFBR2* | 0.00704608 | -1.55352 |
| *DCLK3* | 0.00131114 | 1.85674 |
| *LRRC2* | 0.00232792 | 1.52636 |
| *ABLIM2* | 0.000300094 | 1.54586 |
| *ETNPPL* | 0.00378955 | -1.55655 |
| *PRSS12* | 0.00173976 | 1.57048 |
| *NDST3* | 0.00543777 | 1.5164 |
| *TMEM155* | 0.00791988 | 1.53559 |
| *C4orf45* | 0.00148535 | 1.63451 |
| *TMEM144* | 0.0267798 | -1.57588 |
| *FAM198B* | 0.000414366 | -1.50076 |
| *HHIP* | 0.0142364 | -1.54694 |
| *RANBP3L* | 0.00986125 | -1.54318 |
| *GPR98* | 0.0173903 | -1.5025 |
| *HTR4* | 0.0086221 | 1.50671 |
| *CARTPT* | 0.00297126 | 1.99297 |
| *HCN1* | 0.00757797 | 1.55987 |
| *PTTG1* | 0.00114485 | 1.57475 |
| *SP8* | 0.00258 | 1.81455 |
| *TNFRSF11B* | 0.0131275 | -1.65514 |
| *KCNV1* | 0.0104966 | 1.59428 |
| *ZMAT4* | 0.000584953 | 1.76987 |
| *NKX6-3* | 0.000959325 | 1.70493 |
| *SLC7A3* | 0.0033698 | 1.52706 |
| *LRFN5* | 0.00259449 | 1.52136 |
| *SLC16A9* | 0.00928996 | -1.61937 |
| *DRGX* | 0.0479231 | 1.51731 |
| *RET* | 0.000387653 | 1.69588 |
| *VWA2* | 0.00902291 | -1.50986 |
| *CLMN* | 0.00958958 | -1.5315 |
| *NELL1* | 0.00341032 | 1.6847 |
| *C1QL3* | 0.024328 | 1.70677 |
| *KCNC2* | 0.00781848 | 1.55227 |
| *SVOP* | 0.0100873 | 1.56982 |
| *LRTM2* | 0.00578769 | 1.52231 |
| *PRKCB* | 0.00184085 | 1.50772 |
| *CLEC4E* | 0.0424276 | -1.77261 |
| *SLC38A8* | 0.0135689 | 1.69817 |
| *CHRFAM7A* | 0.000249847 | 1.75436 |
| *TMPRSS5* | 0.0184352 | -1.66789 |
| *PLIN1* | 0.0146368 | -1.53726 |
| *SCNN1G* | 0.00064116 | 1.82077 |
| *CACNG2* | 0.000471101 | 1.63194 |
| *TAC3* | 0.00168395 | 1.85067 |
| *GREM1* | 0.0460318 | -1.52409 |
| *LOXHD1* | 0.00220957 | 1.68189 |
| *LY6D* | 0.0153847 | 1.57245 |
| *KLK6* | 0.0331353 | -1.53133 |
| *ANGPTL4* | 0.010444 | -1.6445 |
| *TBX10* | 0.00395136 | 1.60115 |
| *ENTPD3* | 0.000133427 | 1.81812 |
| *PNOC* | 0.000461984 | 2.13651 |
| *PTF1A* | 0.000949383 | 1.65586 |
| *FAM107A* | 0.00315887 | -1.61106 |
| *SDPR* | 0.0106895 | -1.5133 |
| *CRYGA* | 0.0173452 | 1.67801 |
| *WFDC13* | 0.0291834 | 1.63913 |
| *HTR1E* | 0.00938676 | 1.53472 |
| *FSTL5* | 0.00249265 | 1.52883 |
| *PARM1* | 0.0016999 | 1.70553 |
| *RAB3B* | 0.0177456 | 1.5333 |
| *CLIC4* | 0.00112958 | -1.51831 |
| *GPR183* | 0.0138169 | -1.70402 |
| *MUC15* | 0.0162938 | 1.74177 |
| *ANTXR1* | 0.000342236 | -1.66406 |
| *DRD5* | 0.00693666 | 1.56863 |
| *MT1E* | 0.00568551 | -1.51243 |
| *P2RY1* | 0.0130879 | -1.65496 |
| *FRMPD4* | 0.00228193 | 1.60581 |
| *YWHAG* | 0.00266656 | 1.53434 |
| *FOS* | 0.0249927 | -1.664 |
| *VSTM2A* | 0.00142602 | 1.75594 |
| *GPR37* | 0.0139357 | -1.60468 |
| *SDR16C5* | 0.000471101 | 1.7301 |
| *TRH* | 0.00928996 | 1.5798 |
| *KCNG3* | 0.00247509 | 1.68697 |
| *SOSTDC1* | 0.00632923 | 1.65032 |
| *KCNK3* | 0.000968729 | 1.51337 |
| *KRT19* | 0.00391205 | -1.76911 |
| *KSR2* | 0.00105979 | 1.50176 |
| *ENC1* | 0.0116985 | 1.59432 |
| *AQP4* | 0.00175113 | -1.84247 |
| *CALB2* | 0.000193609 | 2.16189 |
| *SPTLC3* | 0.0120751 | -1.87545 |
| *ABCG4* | 0.00049124 | 1.59818 |
| *FNDC9* | 0.00206738 | 1.55409 |
| *FADS6* | 0.00104286 | 1.62929 |
| *BNC2* | 0.022807 | -1.52103 |
| *TNFRSF10C* | 0.00301527 | -1.72051 |
| *TLR1* | 0.0061807 | -1.61696 |
| *VWC2L* | 0.0025262 | 1.54043 |
| *GALNTL6* | 0.00274228 | 1.52499 |
| *LINGO2* | 0.00708688 | 1.57306 |
| *UGT8* | 0.00958958 | -1.58345 |
| *WDR49* | 0.0204134 | -1.62236 |
| *GPR149* | 0.00304654 | 1.99632 |
| *CHRNA7* | 0.000300094 | 1.61751 |
| *NRIP3* | 0.00135872 | 1.63188 |
| *P2RY2* | 0.00279421 | -1.6565 |
| *C1orf172* | 0.013789 | 1.65973 |
| *EIF4E1B* | 0.000824647 | 1.53883 |
| *CREG2* | 0.00377846 | 1.56039 |
| *KLHL38* | 0.00681453 | 1.64655 |
| *NUPR1* | 0.00416591 | -1.51755 |
| *CIDEA* | 0.00301008 | 1.52123 |
| *DMRTA1* | 0.00181249 | -1.572 |
| *FOXC2* | 0.049071 | -1.70383 |
| *TCERG1L* | 0.00932504 | 1.52289 |
| *RIMKLA* | 0.000538322 | 1.50606 |
| *KCNA3* | 0.00315887 | 1.60978 |
| *GJD4* | 0.000724194 | 1.65008 |
| *C8orf47* | 0.00113387 | 1.61252 |
| *OR2T8* | 0.0377922 | 1.51107 |
| *CD163* | 0.0384019 | -1.93034 |
| *C12orf68* | 0.0040193 | 1.5576 |
| *ZNF366* | 0.00681786 | -1.79565 |
| *HTR1A* | 0.0200895 | 1.52116 |
| *OTOS* | 0.00547035 | -1.64472 |
| *C13orf45* | 0.00630547 | 1.6233 |
| *MSC* | 0.00604071 | 1.52473 |
| *TRIML2* | 0.0173072 | 1.5205 |
| *ALOXE3* | 0.000849045 | 1.59457 |
| *AKAP5* | 0.00539701 | 1.53979 |
| *SERTM1* | 0.0227827 | 1.58907 |
| *CHRM4* | 0.000906368 | 1.81311 |
| *CXCR2* | 0.0498286 | -1.8957 |
| *GREM2* | 0.000505016 | 1.83797 |
| *PENK* | 0.0250457 | 1.53799 |
| *C4orf50* | 0.0135689 | 1.54037 |
| *UTS2R* | 0.00189009 | 1.67296 |
| *AC026703.1* | 0.000308101 | 2.00941 |
| *RTKN2* | 0.00337306 | 1.51101 |
| *TMEM30B* | 0.0472859 | -1.68985 |
| *ZAR1* | 0.000584953 | 2.03755 |
| *GABRG3* | 0.00856561 | 1.59683 |
| *TSHZ2* | 0.000130196 | 1.56808 |
| *RGS6* | 0.000821001 | 1.62026 |
| *PLCXD3* | 0.000130196 | 1.88195 |
| *GPIHBP1* | 0.0275919 | -1.7213 |
| *FREM3* | 0.00200184 | 2.1569 |
| *GRIN2A* | 0.00341504 | 1.58835 |
| *SSTR3* | 0.00150871 | 1.5741 |
| *NPBWR1* | 0.00279068 | 2.14131 |
| *KCTD8* | 0.000192865 | 1.51513 |
| *TUBA8* | 0.000693724 | 1.6778 |
| *SCN5A* | 0.000959325 | 1.62326 |
| *PABPC1L2B* | 0.00154558 | 1.52333 |
| *NCMAP* | 0.0136552 | -1.60871 |
| *TUSC5* | 0.0259029 | 1.54085 |
| *WBSCR17* | 0.00203327 | 1.58154 |
| *SPPL2C* | 0.00142645 | 1.78179 |
| *HS6ST3* | 0.0163339 | 1.50042 |
| *SYN3* | 0.000342236 | 1.64971 |
| *C11orf87* | 0.00605864 | 1.60872 |
| *KCNQ5* | 0.00486782 | 1.59904 |
| *RTN4RL1* | 0.00189009 | 1.54666 |
| *CALHM1* | 0.00255895 | 1.61821 |
| *KRT5* | 0.00260956 | 2.08638 |
| *AGBL4* | 0.0021206 | 1.51631 |
| *FFAR4* | 0.0018947 | 1.5675 |
| *SLC51B* | 0.0267798 | -1.5124 |
| *CYP4F12* | 0.0276891 | -1.73782 |
| *SOWAHB* | 0.0096523 | 1.73906 |
| *PABPC1L2A* | 0.000471101 | 1.63417 |
| *RGS7BP* | 0.0170543 | 1.52338 |
| *MYT1L* | 0.00285879 | 1.53188 |
| *GPR141* | 0.0355463 | -1.62258 |
| *FPR3* | 0.0202424 | -1.67691 |
| *HAPLN4* | 0.000851586 | 1.975 |
| *THSD4* | 0.00801878 | -1.57685 |
| *GABRD* | 0.00632923 | 1.55431 |
| *LIN28B* | 0.00434673 | 1.50521 |
| *COL4A5* | 0.0143578 | -1.57618 |
| *SELL* | 0.0260323 | -1.59875 |
| *CERKL* | 0.00104384 | 1.70254 |
| *SERPINA5* | 0.0395624 | -2.02583 |
| *PAQR9* | 0.00312518 | 1.54864 |
| *ADRB3* | 0.00114504 | 1.87702 |
| *FAM78B* | 0.000342236 | 1.53559 |
| *ASTL* | 0.0333162 | 1.56049 |
| *GJB3* | 0.0112123 | 1.72304 |
| *LITAF* | 0.00272825 | -1.50544 |
| *IL1RAPL2* | 0.00737257 | 1.91487 |
| *OR14I1* | 0.0091876 | 1.68814 |
| *SH2D5* | 0.00253174 | 1.60572 |
| *SERPINA3* | 0.0139377 | -2.28749 |
| *FGF16* | 0.0065032 | 1.71941 |
| *ARL9* | 0.00116501 | 1.59263 |
| *SPTSSB* | 0.00740402 | 1.53894 |
| *ADH1B* | 0.00573873 | -1.81467 |
| *SCN8A* | 0.00183878 | 1.57702 |
| *DTHD1* | 0.0181756 | -1.75624 |
| *C6orf141* | 0.0228634 | 1.68002 |
| *C17orf102* | 0.00314874 | 1.76639 |
| *LRP10* | 0.00110743 | -1.50153 |
| *C16orf47* | 0.00781848 | 1.58853 |
| *SULT1C4* | 0.0111782 | -1.50173 |
| *FAM19A2* | 0.000724194 | 1.77082 |
| *EGFL6* | 0.046341 | 1.67216 |
| *RASSF9* | 0.0173452 | -1.88094 |
| *CCDC152* | 0.0122367 | -1.53651 |
| *CR1* | 0.0212019 | -1.79713 |
| *GPRIN2* | 0.00183881 | 1.55437 |
| *COL5A2* | 0.00465985 | 1.52616 |
| *VWA7* | 0.00114572 | 1.64218 |
| *FOXD4L6* | 0.000193641 | 1.96452 |
| *ATP10A* | 0.00388238 | -1.50788 |
| *TRIM71* | 0.000303653 | 1.61864 |
| *KRT222* | 0.00494218 | 1.5186 |
| *IQCJ* | 0.00388238 | 1.60996 |
| *FAM188B2* | 0.00632004 | 1.8725 |
| *AC025287.1* | 0.0432615 | 1.60265 |
| *VSTM5* | 0.00142321 | 1.61744 |
| *C10orf105* | 0.0102832 | -1.8048 |
| *C12orf71* | 0.0015758 | 1.65138 |
| *RP11-477N12.3* | 0.000300094 | 2.32793 |
| *AC129492.6* | 0.00167506 | 1.56653 |
| *SCRT2* | 0.000406932 | 1.89097 |
| *SIAH3* | 0.000179969 | 1.9022 |
| *SP9* | 0.000130196 | 1.96941 |
| *HTR5A-AS1* | 0.00181977 | 1.72955 |
| *AC092675.3* | 0.0350349 | -1.61827 |
| *C4B* | 0.00189009 | -1.74893 |
| *AC079341.1* | 0.00614467 | 1.64134 |
| *CTB-58E17.5* | 0.000342401 | 1.58757 |
| *C18orf42* | 0.00313114 | 2.04569 |
| *SCAND3* | 0.000767958 | 1.56949 |
| *CRYBG3* | 0.0114673 | -1.61927 |
| *AC012215.1* | 0.00997666 | 1.56227 |
| *SHISA8* | 0.00050535 | 1.83209 |
| *RP11-383H13.1* | 0.00175113 | 1.52897 |
| *ANKRD34C* | 0.0140031 | 1.53523 |
| *AC018816.3* | 0.007958 | 1.50902 |
| *GABARAPL3* | 0.012594 | 1.53508 |
| *AQP1* | 0.00876776 | -1.95894 |
| *PLCXD2* | 0.00298296 | 1.62966 |
| *C4A* | 0.00226846 | -1.72506 |
| *AQP1* | 0.00671923 | -1.90895 |
| *SEPP1* | 0.00721197 | -1.50891 |
| *OC90* | 0.0159917 | 1.61434 |
| *SLC10A5* | 0.000191301 | 1.54515 |
| *RP11-481A20.11* | 0.00941947 | 1.527 |
| *CCDC177* | 0.00158776 | 1.63857 |
| *MTRNR2L10* | 0.0178099 | 2.13686 |
| *MTRNR2L1* | 0.0323876 | 2.93847 |
| *MGAM* | 0.00808822 | -1.54291 |
| *RP11-162P23.2* | 0.000580548 | -1.55335 |
| *CLEC5A* | 0.0262597 | -1.68495 |
| *RP11-497E19.2* | 0.00742176 | 1.65403 |
| *RP11-131H24.4* | 0.0119005 | 1.53411 |
| *RP11-133K1.2* | 0.0014809 | 1.57123 |
| *C6orf229* | 0.00948487 | 1.69126 |
| *TPBGL* | 0.00684677 | 1.50226 |
| *CTD-2370N5.3* | 0.0175327 | -1.57092 |
| *AC011897.1* | 0.00119631 | 1.51542 |
| *AL626787.1* | 0.000527235 | 1.5979 |
| *CCDC177* | 0.000562353 | 1.70063 |
| *AL161784.1* | 0.00990219 | -1.57482 |
| *AP002956.1* | 0.00728867 | 1.53997 |
| *AC018470.1* | 0.000570628 | 1.95128 |
| *AP000889.3* | 0.00508593 | 1.59235 |
| *AL358113.1* | 0.00175113 | -1.50341 |
| *MTRNR2L6* | 0.0174724 | 1.85554 |
| *GRIN2B* | 0.00211965 | 1.64837 |
| *RP11-986E7.7* | 0.01464 | -2.28278 |

**Table S3. DEGS in CER.** Positive fold change represents higher gene expression in SS than TT. Negative fold change represents lower gene expression in SS than TT.

| **Gene name** | **FDR** | **Fold Change** |
| --- | --- | --- |
| *CACNG3* | 0.0138067 | 2.03429 |
| *PAX2* | 0.000866182 | 1.8121 |
| *LBX1* | 0.0469877 | 1.84798 |
| *SORCS3* | 0.0469877 | 1.75029 |
| *GRM2* | 0.000866182 | 1.74021 |
| *SLC6A5* | 0.00495647 | 2.74419 |
| *CPLX4* | 0.000866182 | 2.6622 |

**Table S4. Overlapping genes between TCX and CER based on top 500 genes with |FC| ≥ 1.2 ranked by unadjusted p value.**

| **Gene name** | **TCX**  **P value** | **TCX**  **FDR** | **TCX**  **Fold change** | **CER**  **P value** | **CER FDR** | **CER**  **Fold change** |
| --- | --- | --- | --- | --- | --- | --- |
| *RPP25* | 8.1637E-08 | 0.0001302 | 1.45505 | 0.00547275 | 0.519357 | 1.22532 |
| *BTBD11* | 8.507E-08 | 0.0001302 | 1.85957 | 5.80493E-05 | 0.079726 | 1.83012 |
| *GRIN2D* | 1.742E-07 | 0.0001302 | 1.40952 | 0.0047158 | 0.494466 | 1.26632 |
| *IGSF3* | 2.2605E-07 | 0.0001467 | 1.65359 | 0.0417265 | 0.768656 | 1.20908 |
| *SIAH3* | 3.7343E-07 | 0.00018 | 1.9022 | 0.0477509 | 0.768656 | 1.45155 |
| *SLC32A1* | 1.7932E-06 | 0.0003314 | 2.24779 | 0.077422 | 0.824888 | 1.38636 |
| *LGI2* | 1.9258E-06 | 0.0003395 | 1.48684 | 0.0005089 | 0.177452 | 1.50089 |
| *CHRNA2* | 2.1111E-06 | 0.0003422 | 2.10659 | 0.0286895 | 0.707516 | 1.52128 |
| *SYN3* | 2.1592E-06 | 0.0003422 | 1.64971 | 0.0330237 | 0.724088 | 1.26304 |
| *GJD2* | 2.3313E-06 | 0.0003422 | 1.9986 | 0.000157813 | 0.124539 | 1.77353 |
| *SCRT2* | 3.5463E-06 | 0.0004069 | 1.89097 | 0.0331983 | 0.724088 | 1.27805 |
| *BEAN1* | 5.788E-06 | 0.0004842 | 1.30448 | 0.00029669 | 0.125151 | 1.3083 |
| *GREM2* | 6.1974E-06 | 0.000505 | 1.83797 | 0.060958 | 0.799137 | 1.24821 |
| *GAD1* | 1.391E-05 | 0.0007425 | 1.90105 | 0.0212663 | 0.685816 | 1.44691 |
| *ACTC1* | 1.397E-05 | 0.0007433 | 1.48549 | 0.000666535 | 0.192193 | 1.32555 |
| *CYGB* | 1.5107E-05 | 0.000768 | 1.45156 | 0.0098722 | 0.596772 | 1.2599 |
| *GAD2* | 1.6436E-05 | 0.0007788 | 2.13815 | 0.0252224 | 0.693585 | 1.48965 |
| *PARP4* | 1.8014E-05 | 0.000821 | -1.46153 | 0.0136953 | 0.624407 | -1.21072 |
| *MYO16* | 1.9111E-05 | 0.0008308 | 1.42005 | 0.00038275 | 0.143474 | 1.37794 |
| *ALOXE3* | 1.9933E-05 | 0.000849 | 1.59457 | 0.0284553 | 0.705123 | 1.24783 |
| *MARCH4* | 2.5251E-05 | 0.0009593 | 1.63965 | 0.000300482 | 0.125151 | 1.48226 |
| *DUSP1* | 2.9597E-05 | 0.0010429 | -1.58732 | 0.0762951 | 0.824888 | -1.20928 |
| *MCHR1* | 3.0287E-05 | 0.0010438 | 1.74646 | 0.00014349 | 0.124497 | 1.70197 |
| *DPT* | 3.0517E-05 | 0.0010438 | 1.4358 | 0.077649 | 0.824888 | 1.27765 |
| *HIPK4* | 3.873E-05 | 0.001165 | 1.5772 | 0.0475426 | 0.768656 | 1.22865 |
| *AC011897.1* | 4.0806E-05 | 0.0011963 | 1.51542 | 0.00178082 | 0.333321 | 1.37267 |
| *SCGN* | 4.5817E-05 | 0.0012766 | 1.62972 | 0.0100458 | 0.601873 | 1.54963 |
| *TLR4* | 4.6319E-05 | 0.0012818 | -1.70536 | 0.102081 | 0.870087 | -1.20375 |

**Table S5**. **Enrichment of modules for their respective DEG signatures.**

| **Module** | **Tissue** | **Enrichment FDR** | **# overlap between module and DEG** |
| --- | --- | --- | --- |
| black | TCX | 0.012224 | 32 |
| blue | TCX | 1 | 65 |
| brown | TCX | 0.0026 | 46 |
| cyan | TCX | 1 | 1 |
| darkgreen | TCX | 1 | 2 |
| darkgrey | TCX | 1 | 0 |
| darkred | TCX | 0.716745 | 5 |
| darkturquoise | TCX | 1 | 0 |
| green | TCX | 1 | 2 |
| greenyellow | TCX | 1 | 12 |
| grey60 | TCX | 1 | 0 |
| lightcyan | TCX | 1 | 0 |
| lightgreen | TCX | 1 | 1 |
| lightyellow | TCX | 1 | 6 |
| magenta | TCX | 1 | 2 |
| midnightblue | TCX | 1 | 1 |
| pink | TCX | 1 | 5 |
| purple | TCX | 1 | 4 |
| red | TCX | 1 | 0 |
| royalblue | TCX | 1 | 0 |
| salmon | TCX | 1 | 15 |
| tan | TCX | 1 | 4 |
| turquoise | TCX | 8.22E-52 | 346 |
| yellow | TCX | 1 | 0 |
| black | CER | 1 | 6 |
| blue | CER | 1 | 42 |
| brown | CER | 1 | 15 |
| green | CER | 1 | 3 |
| greenyellow | CER | 1 | 1 |
| magenta | CER | 1 | 1 |
| pink | CER | 1.11E-12 | 29 |
| purple | CER | 2.01E-49 | 51 |
| red | CER | 1 | 4 |
| salmon | CER | 1 | 0 |
| tan | CER | 1.15E-72 | 57 |
| turquoise | CER | 1 | 14 |
| yellow | CER | 1 | 1 |

**Table S6. Significant modules identified in the TCX and CER matched cases.**

| **Module** | **Correlation** | **P value** | **Top GO** | **FDR** | **Tissue** |
| --- | --- | --- | --- | --- | --- |
| turquoise | 0.48 | 1.00E-05 | synaptic transmission | 8.67E-45 | TCX |
| red | 0.41 | 3.00E-04 | intracellular membrane-bounded organelle | 0.018288 | TCX |
| greenyellow | 0.37 | 0.001 | NADH dehydrogenase activity | 6.32E-11 | TCX |
| black | 0.34 | 0.003 | mitotic spindle organization | 1 | TCX |
| darkgreen | 0.25 | 0.04 | transcription cofactor activity | 0.104463 | TCX |
| midnightblue | -0.48 | 2.00E-05 | enucleate erythrocyte differentiation | 1 | TCX |
| blue | -0.42 | 2.00E-04 | axon ensheathment | 2.31E-05 | TCX |
| white | -0.4 | 4.00E-04 | nucleus | 8.74E-10 | TCX |
| brown | -0.37 | 1.00E-03 | carboxylic acid catabolic process | 1.08E-07 | TCX |
| salmon | -0.32 | 6.00E-03 | extracellular matrix organization | 2.07E-21 | TCX |
| royalblue | -0.27 | 2.00E-02 | innate immune response | 8.93E-06 | TCX |
| tan | 0.37 | 6.00E-04 | synaptic transmission | 0.000121 | CER |
| salmon | -0.27 | 1.00E-02 | immune response | 2.37E-18 | CER |

**Table S7. Significant modules identified in separate disease groups in TCX and CER.**

| **Module** | **Correlation** | **P value** | **Top GO** | **FDR** | **Group** |
| --- | --- | --- | --- | --- | --- |
| turquoise | 0.48 | 0.001 | synaptic transmission | 7.61E-33 | TCX-AD |
| lightgreen | 0.35 | 0.02 | protein binding | 1 | TCX-AD |
| royalblue | -0.55 | 2E-04 | sarcoglycan complex | 1 | TCX-AD |
| lightyellolw | -0.46 | 0.002 | regulation of transcription from RNA polymerase II promoter | 1 | TCX-AD |
| pink | -0.42 | 0.006 | activation of NF-kappaB-inducing kinase activity | 0.93 | TCX-AD |
| brown | -0.41 | 0.007 | axon ensheathment | 0.0007 | TCX-AD |
| midnightblue | -0.4 | 0.008 | DNA binding | 2.34E-06 | TCX-AD |
| yellow | -0.35 | 0.02 | homophilic cell adhesion | 1.51E-08 | TCX-AD |
| red | -0.39 | 0.01 | extracellular region | 2.16E-25 | TCX-AD |
| blue | -0.33 | 0.03 | Nucleus | 2.01E-15 | TCX-AD |
| salmon | -0.32 | 0.04 | nucleic acid binding | 0.01 | TCX-AD |
| yellow | 0.53 | 2E-04 | synaptic transmission | 6.58E-22 | TCX-PSP |
| turquoise | 0.47 | 0.001 | cell signaling | 4.26E-08 | TCX-PSP |
| royalblue | -0.43 | 0.003 | DNA binding | 0.002 | TCX-PSP |
| white | -0.42 | 0.004 | response to wounding | 1 | TCX-PSP |
| darkgreen | -0.41 | 0.005 | cytoplasmic ubiquitin ligase complex | 1 | TCX-PSP |
| greenyellow | -0.39 | 0.009 | cell division | 0.69 | TCX-PSP |
| salmon | -0.37 | 0.01 | cytosolic ribosome | 1.57E-100 | TCX-PSP |
| orange | -0.34 | 0.02 | transcription, DNA-templated | 2.81E-11 | TCX-PSP |
| red | -0.34 | 0.02 | axon ensheathment | 2.96E-06 | TCX-PSP |
| greenyellow | 0.32 | 0.03 | synaptic transmission | 0.004 | CER-AD |
| lightcyan | -0.34 | 0.02 | Immune response | 1.07E-23 | CER-AD |
| darkorange | 0.48 | 8E-04 | synaptic transmission | 0.0006 | CER-PSP |

**Table S8. Significant modules identified in the TCX and CER controls.**

| **Module** | **Correlation** | **P value** | **Top GO** | **FDR** | **Tissue** | |
| --- | --- | --- | --- | --- | --- | --- |
| lightcyan | 0.34 | 0.03 | CHOP-C/EBP complex | 1 | TCX |  |
| brown | -0.36 | 0.02 | carboxylic acid catabolic process | 0.001834 | TCX |  |
